# Applications of Raman spectroscopy in ocular biofluid detection

**DOI:** 10.3389/fchem.2024.1407754

**Published:** 2024-06-10

**Authors:** Zhijun Guo, Miaoli Ma, Sichao Lu, Ying Ma, Yansuo Yu, Qianjin Guo

**Affiliations:** ^1^ Beijing Institute of Petrochemical Technology, Beijing, China; ^2^ Beijing Academy of Safety Engineering and Technology, Beijing, China

**Keywords:** aqueous humor, biomolecules, ocular biofluids, Raman spectroscopy, tear

## Abstract

Ophthalmic and many systemic diseases may damage the eyes, resulting in changes in the composition and content of biomolecules in ocular biofluids such as aqueous humor and tear. Therefore, the biomolecules in biofluids are potential biomarkers to reveal pathological processes and diagnose diseases. Raman spectroscopy is a non-invasive, label-free, and cost-effective technique to provide chemical bond information of biomolecules and shows great potential in the detection of ocular biofluids. This review demonstrates the applications of Raman spectroscopy technology in detecting biochemical components in aqueous humor and tear, then summarizes the current problems encountered for clinical applications of Raman spectroscopy and looks forward to possible approaches to overcome technical bottlenecks. This work may provide a reference for wider applications of Raman spectroscopy in biofluid detection and inspire new ideas for the diagnosis of diseases using ocular biofluids.

## 1 Introduction

Ocular biofluids such as aqueous humor (AH) and tear contain abundant biomolecules, including proteins, lipids, carbohydrates, vitamins, urea, etc., which are usually maintained at a relatively stable level. Ophthalmic and many systemic diseases, such as diabetes, hypertension, microbial infection, and accidental injury, may damage the eyes, resulting in changes in the composition and content of biomolecules in ocular biofluids. Therefore, these biomolecules can be used as disease markers to reveal pathological processes and to diagnose ophthalmic and systemic diseases. Quantitative information on the chemical composition of AH is generally obtained through invasive techniques such as biopsy or needle absorption and *in vitro* detection (Li et al., 2023a). However, these invasive techniques cause discomfort for patients, while leaving wounds that increase the risk of infection. Qualitative and quantitative techniques used for the analysis of tear components include mass spectrometry, high-performance liquid chromatography (HPLC), direct immunofluorescence analysis, polymerase chain reaction, electrophoresis, and the most common enzyme-linked immunosorbent assay. Although these methods can effectively obtain information on tear components, the requirement of large and expensive instruments, and equipment, chemical reagents, or long testing time limits their wide applications (Wu and Zhang, 2007; Filik and Stone, 2008).

Raman spectroscopy (RS), as a vibrational spectroscopy technique, obtains chemical bond information of the analyte molecule by analyzing the inelastically scattered light excited by monochromatic light irradiation on the analyte. It supports quick, label-free, and non-destructive evaluation of biochemical molecules in human cells, tissues, and body fluids at an inexpensive cost, and has been increasingly applied in the biomedical field. Since RS can display rich biomolecule structures and is less affected by water compared to infrared spectroscopy technology, the qualitative and quantitative analysis of Raman-active biomolecules in ocular biofluid using RS has become a promising method for biological component analysis. The application of RS in ocular biofluid detection has attracted a lot of attention and has become a research focus. To obtain highly-sensitive specific signals of the biomolecules, RS combined with confocal techniques (CRS), resonant Raman scattering (RRS), surface-enhanced Raman scattering (SERS), and drop-coating deposition Raman spectroscopy (DCDRS) were proposed and applied in several researches. Due to the potential presence of multiple molecular structural information in the spectrum, the intelligent disease diagnosis method that utilizes the high-dimensional information of the Raman spectrum as features to train models for pattern recognition of disease samples was proposed and has made encouraging progress. This article reviews the applications of RS technology in the detection of biochemical components in AH and tear, then summarizes the current problems encountered for clinical applications of RS and looks forward to possible methods to overcome technical bottlenecks. This review references almost all articles related to Raman detection of ocular biofluid. There are 46 articles retrieved from Google Scholar using keywords such as the combination of “Raman spectroscopy”, “aqueous humor”, and “tear” were referenced. Among all the references, 22 articles were published in the last 5 years.

## 2 Applications of Raman spectroscopy in ocular biofluid detection

### 2.1 Aqueous humor

Aqueous humor is a transparent and clear liquid produced by the ciliary body that fills the anterior and posterior chambers. It has the function of maintaining intraocular pressure, supplying nutrients and oxygen to the eye tissues, such as the cornea and lens, excreting their metabolites, and so on. The AH contains high concentrations of glucose (about 80% of blood glucose (BG)), ascorbic acid, small amounts of protein and lactate, ascorbate, pyruvate, urea, and other metabolites. The stability of the content of these components is crucial for maintaining the normal physiological function of the eyes. When harmful substances or circulatory disorders appear in AH, the normal metabolism of the lens and cornea are endangered, leading to various diseases. Because the anterior chamber is close to the outside of the body and bounded by the transparent cornea, non-invasive detection of aqueous humor components using RS becomes possible. The AH detection using RS has shown great potential in non-invasive monitoring of BG concentration and ophthalmic pharmacokinetic analysis.

#### 2.1.1 Non-invasive monitoring of blood glucose concentration

**FIGURE 1:**
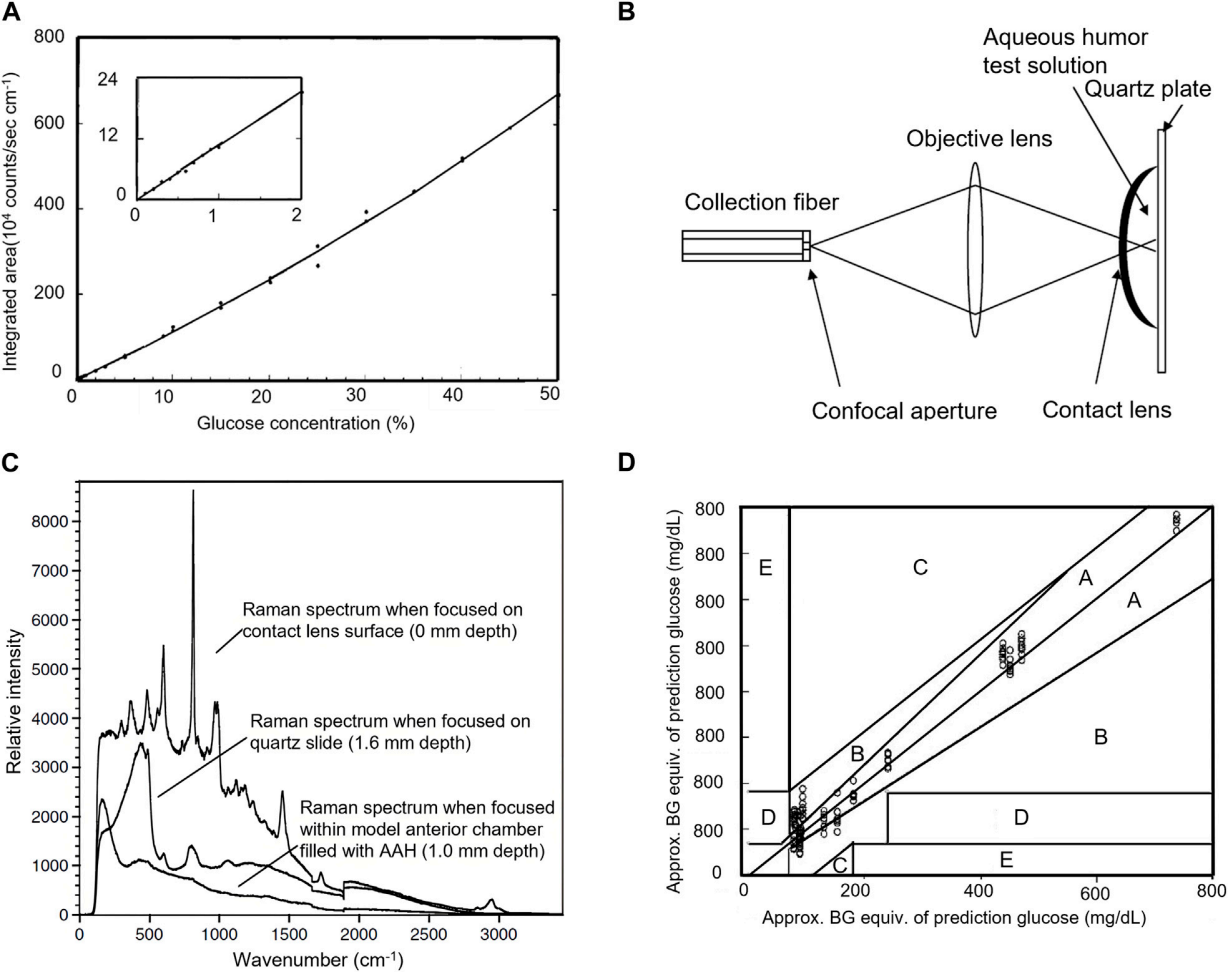
**(A)** Integrated area of glucose doublet as a function of glucose concentration (Wang et al., 1993). **(B)** Confocal collection of Raman spectra from a model anterior chamber and **(C)** the Raman spectra of the aqueous solution confocally collected from a depth of 1.0 mm beneath the surface of the contact lens (Lambert et al., 2002). **(D)** Clarke grid glucose concentrations predicted from spectra of human AH have been approximately converted to BG equivalent concentrations (Lambert et al., 2005).

Plasma is the mother liquor that forms AH, and the electrolytes and other components in AH are roughly the same as those in blood. The glucose concentration in AH is related to BG, and the time lag reflecting BG changes is relatively short (Chan et al., 2022). Since the blood-aqueous barrier filters out most large molecules, AH is a less complex fluid than blood and is more easily accessible optically than blood in the tissue. These characteristics of AH make it a good candidate for evaluating BG and non-invasive monitoring of BG concentration using RS of AH in the anterior chamber has become an attractive target. To clarify the quantitative ability of Raman signature on glucose, Wang et al. compared the water background subtracted Raman spectra of four aqueous solutions of glucose, lactate, ascorbate, and urea, respectively, as well as a mixed solution containing these four components and pyruvate, with those of AH extracted from rabbit (Wang et al., 1993). They found the Raman spectra of the mixed solution and rabbit AH could be obtained by linear sum fitting from known pure metabolite spectra, and the integrated area of glucose doublet was linearly proportional to glucose concentration as shown in Figure 1A. Wicksted monitored the glucose and lactate metabolites within AH extracted from human eyes during cataract surgery and postmortem rabbit eyes *in vitro* using RS (Wicksted et al., 1994; Wicksted et al., 1995). They observed symmetric and antisymmetric stretching modes of methylene and methyl groups associated with glucose and lactate, respectively, and the carbon-nitrogen stretching mode of urea and various amino acids at 1,006 cm^−1^ in both spectra, as well as the second stretching mode characteristic of lactate between the carbon atom and either the carboxylic acid group or the carboxylate ion group at 860 cm^−1^ in the human specimen. To determine the measurement accuracy of glucose at physiological levels, Lambert et al. established a model anterior chamber and acquired Raman spectra of aqueous mixtures of glucose, urea, lactic acid, and ascorbic acid in it using a confocal Raman microscope as shown in Figures 1B, C (Lambert et al., 2002). They further established a multiple regression model using spectral datasets and partial least squares (PLS) analysis to predict glucose concentration with Raman spectra and achieved clinically acceptable predictability, demonstrating the feasibility of non-invasive glucose measurement using RS. Lambert et al. and Pelletier et al. further predicted glucose concentration in human AH *in vitro* using the same method (Lambert et al., 2005; Pelletier et al., 2005). Their experimental result in Figure 1D indicated the accuracy of glucose measurement within the clinically relevant range and the feasibility of determination of glucose levels of patient-derived AH using “artificial AH” calibration solutions when the laser power is safe. In addition to evaluating BG concentration, RS of AH can also be used to diagnose diabetes-related diseases. The biochemical characteristics of oxidative stress in age-related macular degeneration (AMD) and diabetes retinopathy (DR) using RS of AH were studied and the classification of AMD and DR using the spectral characteristics was achieved with a high sensitivity of 100% and high specificity of 100% (Moon et al., 2016).

#### 2.1.2 Ophthalmic pharmacokinetic analysis

**FIGURE 2:**
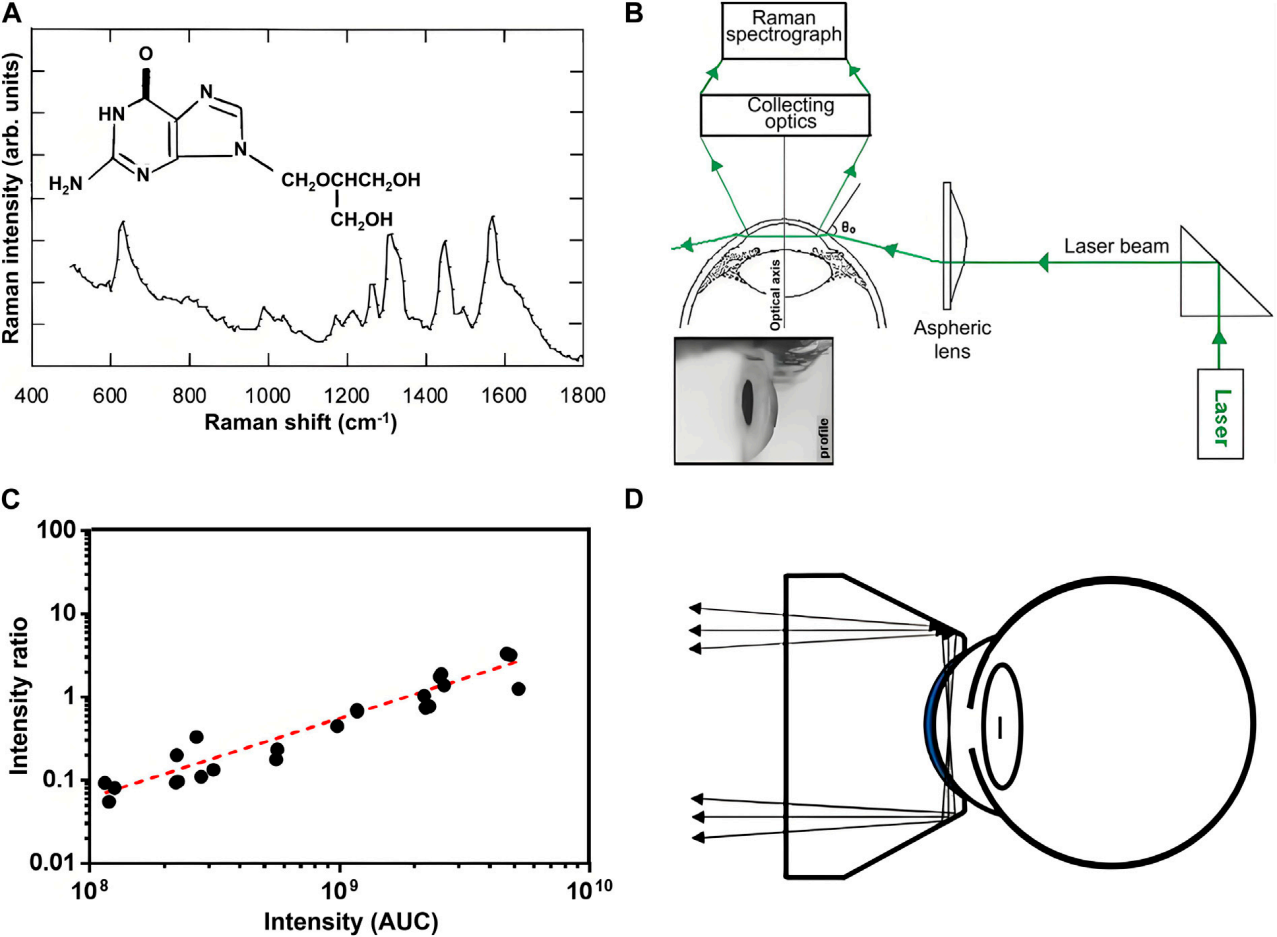
**(A)** Molecular structure of ganciclovir along with its Raman signature (). **(B)** Experimental setup for collecting 90-degree Raman scattering light (Sideroudi et al., 2006). **(C)** Correlation between Raman signal (*y*-axis) and HPLC signal (*x*-axis) of AH from pig eyes submerged in ketorolac dilutions (Bertens et al., 2019). **(D)** Ophthalmic probe for collecting Raman scattering light from AH (Zhang et al., 2021).

Ophthalmic pharmacokinetics studies the functional relationship between the absorption, distribution, and metabolism of ophthalmic drugs with concentration and time, which is crucial for accurately controlling the concentration of ophthalmic drugs to maintain drug levels within the treatment level required for optimal prognosis and to prevent or minimize toxicity associated with a drug overdose. Traditional ophthalmic pharmacokinetic analysis requires the collection of tissue or body fluids for destructive testing to evaluate the time-dependent distribution of drug concentration at sufficient time points. Therefore, a sufficient number of animals are needed, bringing a great challenge to ophthalmic pharmacokinetic analysis. RS, as a non-invasive detection technique, can effectively overcome the above problems and provide a new method for studies of ocular pharmacokinetics. Due to the relatively small number of optically active molecules that can interfere with drug detection in AH, the Raman spectral features of drugs in AH are clearer and easier to identify. This advantage makes the ophthalmic pharmacokinetic analysis based on RS of AH attract a lot of interest. To explore the penitential of RS for quantitative analysis of ophthalmic drugs, Hosseini et al. studied Raman characteristics of different concentrations of ganciclovir in the eyes of anesthetized rabbits *in vivo* by using a confocal Raman scattering setup as shown in Figure 2A (Hosseini et al., 2002). The intensity of the Raman signal was linearly correlated with the local concentration of drugs, indicating RS offers an effective non-invasive tool for evaluating the local concentration of ganciclovir in the anterior chamber. Hosseini et al. further detected ceftazidime and amphotericin B (AmB) in rabbit AH using the same method and determined the Raman signatures of the drugs (Hosseini et al., 2003). To reduce the deposited power on the cornea and enhance the Raman signal of the drug, La et al. used RRS at 406.7 nm to measure AmB concentrations in AH of a rabbit after 5 days of intravenous AmB administration (La et al., 2006). They confirmed the measurement accuracy using RRS by comparing the results to those measured by HPLC. To reduce direct exposure of basic eye tissue to the laser beam while improving the signal-to-noise ratio of the Raman spectrum, Sideroudi et al. constructed an experimental setup as shown in Figure 2B to collect 90-degree Raman scattering light (Sideroudi et al., 2006). With that setup, Raman signatures of low concentrations (close to the minimum inhibitory concentration) of ceftazidime (0.9 mg/mL), AmB (9 mg/mL), and glucose (2 mg/mL, which is close to the early pathological levels of patients with diabetes) separately injected in the anterior chamber of porcine eyes were detected *in vitro*. Sideroudi et al. also developed an RS-based PLS chemometric algorithm to predict the concentration of ciprofloxacin in an artificial anterior chamber over the range from 0 to 1 mg/mL with a correlation coefficient *R* ^2^ = 98.4% and an RMS error of prediction equal to 41 μg/mL (Sideroudi et al., 2007). Bertens et al. detected the concentration of ketorolac tromethamine in AH of enucleated pig eyes and living rabbit eyes after 4 weeks of medication using both CRS and HPLC Bertens et al. (2019). The results indicated RS could detect ketorolac in enucleated pig eyes over a wide concentration range as shown in Figure 2C but was not sensitive enough to detect the drug in living rabbit eyes. To improve Raman intensity as well as the safety of detection, Zhang et al. designed a dark-field ophthalmic contact probe for collecting Raman light from AH as shown in Figure 2D, and detected the phenylephrine HCl in an artificial eye mode with the probe (Zhang et al., 2021). The results indicated great improvement in misalignment tolerance and signal-to-noise ratio at the phenylephrine HCl 1,002 cm^−1^ peak were achieved. The detectable phenylephrine HCl concentration is down to 0.1% and the linear fit between the drug concentration and Raman peak intensity, *R* ^2^ reaches 0.996.

### 2.2 Tear

Human tears are a transparent, slightly milky white aqueous mixture secreted by the lacrimal gland and conjunctival goblet cells. Their functions include keeping the eyes moist, protecting them from injury and infection, flushing waste and small particles, and providing nutrition for the eyes, etc. The composition of tears is extremely complex, consisting of water, protein, lipids, metabolites, glucose, electrolytes, etc. These components are closely related to maintaining eye health and normal function, and changes in their content may indicate the pathogenesis of ophthalmic and systemic diseases. Compared to other human biofluids such as blood, sweat, and urea, tear fluids can be collected easily and noninvasively with capillary tubes, making it a good choice for biochemical component analysis. Due to the advantages of label-free and fast detection, RS in the applications of the characterization of tear components and disease diagnosis has become continuous research hotspots.

#### 2.2.1 Characterization of tear components

**FIGURE 3:**
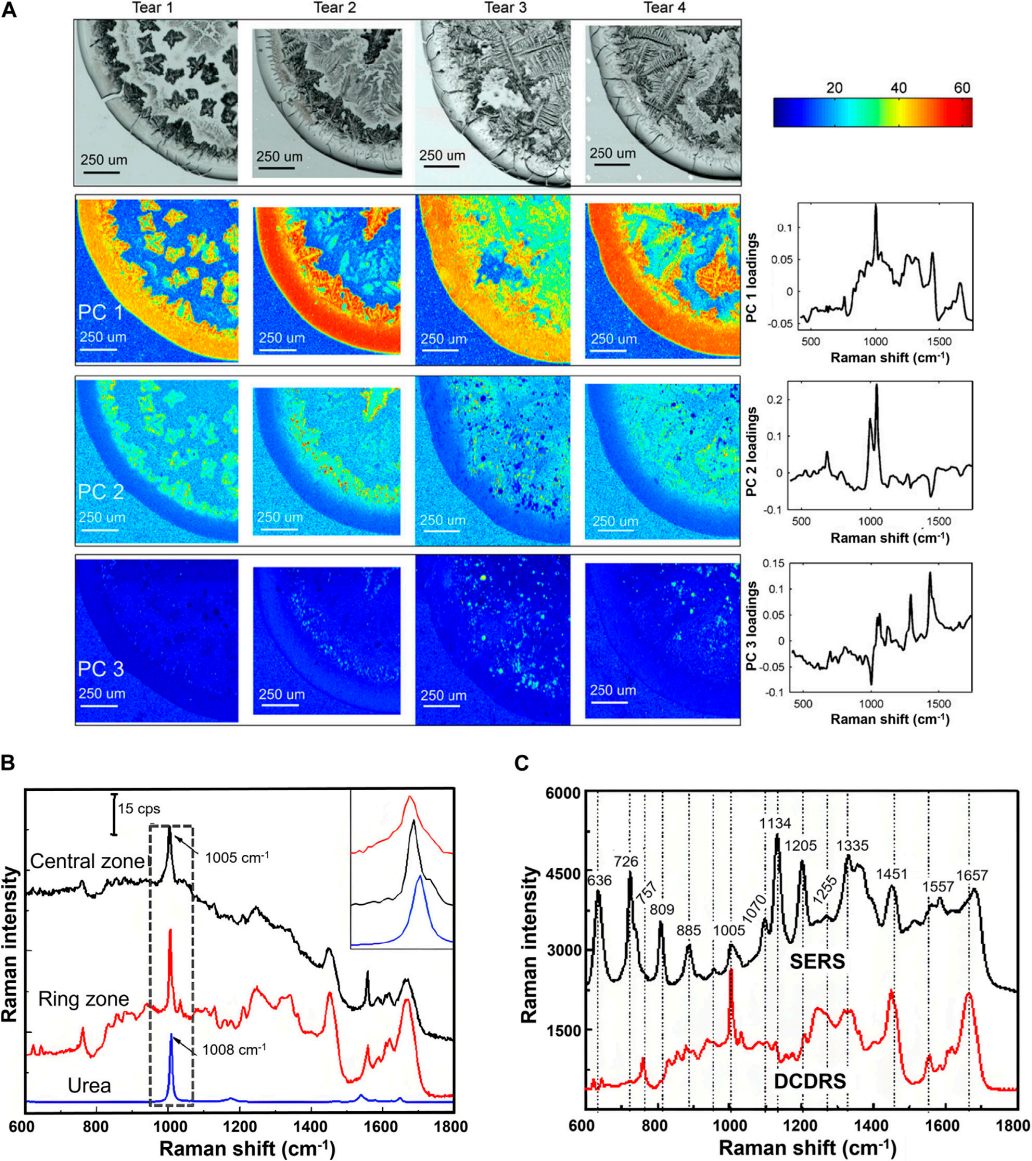
**(A)** White light images and selected false color PC score maps of the four tear samples (Filik and Stone, 2008). **(B)** Raman spectra of the central zone, ring zone, and the Raman spectra of urea and **(C)** comparison between the DCD Raman spectrum and SER spectrum of tears (Hu et al., 2014).

Tears contain hundreds of proteins, including albumin, lactoferrin, lysozyme, etc. The concentration of these proteins as well as other components such as uric acid (UA) and glucose in tears are related to various eye and systemic diseases such as glaucoma, dry eye, thyroid-associated orbitopathy, diabetes mellitus, or cancer (von et al., 2013). Therefore, the characterization of tear components is of great significance for revealing disease markers and analyzing pathological processes. Since the concentration of tear components are usually below the detection limit of RS, SERS based on the plasma resonance effect, and DCDRS based on the coffee ring effect were proposed to amplify the Raman signal. To clarify the best method for tear detection, Reyes-Goddard et al. studied the repeatability and sensitivity of Raman spectra of phenylalanine, tryptophan, lysozyme solutions, and artificially synthesized tear films using SERS based on silver sol, silver electrode, gold film, and silver mirror, respectively (Reyes-Goddard et al., 2003). They found the SERS based on silver mirror had the best spectral repeatability and was hopeful for detecting human tear components. Considering the levels of biochemical components in non-reflective and yawn-reflex secreted tears may differ, Filik et al. analyzed the Raman spectra of these two kinds of teardrops as well as their drop-coating depositions (DCDs) and predicted protein concentrations using standard solution calibration techniques (Filik and Stone, 2008). The signals of protein and urea were observed in all samples, and the DCD technique can effectively improve the spectral signal-to-noise ratio of tear components. The principal component analysis (PCA) on the Raman spectra of dried teardrop is shown in Figure 3A. It was concluded the sample contained proteins, lipids, urea, and bicarbonate, and the uneven distributions of these components were related to their relative solubility and concentration. Further study of the protein composition of human tear fluid using centrifugal filters and DCDRS demonstrated the spectra of tear proteins with different centrifugal masses were different, confirming that the detected signal did not come from a single protein (Filik and Stone, 2009). The radial variation of the DCD Raman spectrum of the ring region indicated the urea and bicarbonate signals become stronger towards the inner edge of the ring, and the tryptophan side chain and amide III bands become stronger towards the outer edge. To understand the differences in detection results between DCDRS and SERS, Raman spectra of human tears obtained using these two methods are systematically compared (Figures 3B, C) (Hu et al., 2014). It was found the main contribution of DCDR spectra in the deposition area of the coffee ring came from high-abundance proteins, including lysozyme, lactoferrin, and albumin, and the main contribution of tear SER spectra came from low abundance UA and hypoxanthine. The combination of these two technologies could provide multi-parameter information for clinical tear systematic analysis. To improve the quantitative ability of RS for tear components, Narasimhan et al. employed a flexible metasurface in a practical broadband SERS to detect human tear UA within its physiological concentration range (25–150 μM) and developed a multifunctional scleral lens made on parylene films with a small sensing region of SERS to detect lysozyme and lactoferrin in DCD of teardrops (Narasimhan et al., 2020; Narasimhan et al., 2023). The detections showed excellent correlation with commercial enzyme-based assays. Lien et al. achieved a detection limit of 25 μM UA in artificial tears using high-sensitive SERS based on special substrates utilizing ZnO nanocages and yolk-shell-structured plasmonic nanomaterials (Lien et al., 2024). Tear is a potential alternative to blood for glucose monitoring because of the correlation between glucose levels in tears and blood. Lee et al. developed an SERS contact lens for glucose sensing in human tears and achieved the limit of glucose detection of 211 nM in the concentration range of 500 nM^−1^ mM (Lee et al., 2021). Cui et al. designed a flexible SERS substrate composed of gold nanoparticles (AuNPs) and two-dimensional MXene Ti3C2TX nanosheets to enhance the sensitivity of the Raman spectrum of glucose in human tears and achieved a detection limit of 0.39 μM within a linear range of 1–50 μM (Cui et al., 2021).

Tear components can also provide valuable information for pharmacokinetics studies and the development of contact lenses. Since tear concentrations of drugs are good indicators for therapeutic drug monitoring in blood, the characterization of drug content in tears using RS provides a new approach for pharmacokinetic studies. Yokoyama et al. detected 1 μg/mL sodium valproic acid in aqueous solution using DCDRS, demonstrating the high sensitivity of the Raman spectrum in monitoring the concentration of valproic acid in tears (Yokoyama et al., 2013). The use of contact lenses (CLs) often causes inflammatory responses in the eye since it alters the natural arrangement and functions of tear film. To reveal the interaction between CL and tear, biochemical changes in the tears of the same individual before and after wearing hydrogel and silicon hydrogel CLs were studied using DCDRS (Capaccio et al., 2019). The experimental results show an alteration of the relative concentrations of proteins and lipids in both of the analyzed cases, indicating the ability of RS to evaluate the changes in tears induced by CLs.

#### 2.2.2 Diagnosis of diseases

**FIGURE 4:**
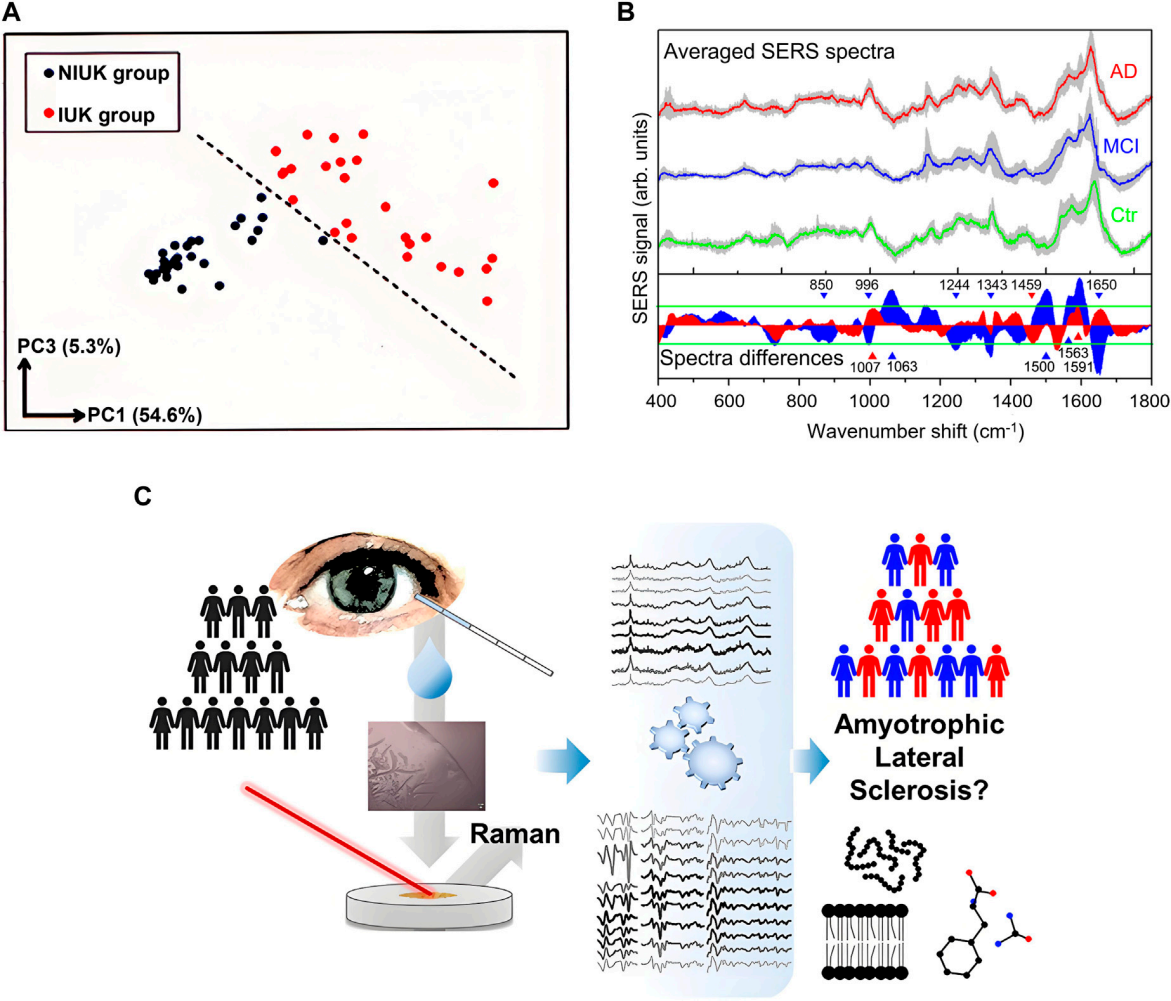
**(A)** Discrimination of the two groups through PC1 and PC3 by PCA (Kuo et al., 2011). **(B)** Averaged SERS spectra and spectra difference of tear samples from Ctr (green line), MCI (blue line), and AD (red line) (Cennamo et al., 2020). **(C)** Discrimination of amyotrophic lateral sclerosis using tear Raman spectra (Ami et al., 2021).

Although tears contain multiple potential disease marker molecules, the complexity and low concentration of tear components, as well as limited prior knowledge of RS of potential biomarkers, make it difficult to directly quantify these contents through spectral features in most cases, especially when the difference between the spectra from patients and normal ones is difficult to perceive intuitively. Fortunately, this problem may be solved by multivariate analysis and machine learning. Especially the analysis of the high-dimensional spectral data via machine learning has become a powerful tool for disease diagnosis and demonstrated a promising prospect. To explore the potential of RS of tears in the diagnosis of conjunctivitis caused by herpes simplex virus (HSV), Reyes-Goddard et al. acquired SERS spectra of virus particles in tear films, synthetic tear (ST), a mixture of ST and transport media (TM), and a mixture of ST, TM, and heat-denatured HSV after drying on silver mirror and gold film substrates (Reyes-Goddard et al., 2008). They constructed datasets using multi-point Raman spectra obtained from scanning dried depositions and classified the samples using linear discriminant analysis (LDA). The sensitivity and specificity for silver mirror reaction were 75.5% ± 13.8%, and 77.3% ± 8.3%, respectively, and for gold film were 75.5% ± 5.9% and 78.3% ± 6.2%, respectively. Choi et al. investigated the feasibility of DCD-SERS of tears in the clinical diagnosis of adenoviral conjunctivitis (Choi et al., 2014). By comparing Raman spectra of normal and diseased tears, three adenovirus-specific DCD-SERS bands, including an amide III β-sheet at 1,242 cm^−1^, a C-H deformation in proteins at 1,342 cm^−1^, and a C-H deformation in DNA/RNA, proteins, lipids, and carbohydrates at 1,448 cm^−1^, were identified as biomarkers of adenovirus. The classification results using the biomarker of the logarithm of I_1242_/I_1342_, PCA, and multi-Gaussian peak fitting of the Raman spectrum showed all three methods had the potential to detect adenoviruses, but the PCA performed the best. Kuo et al. analyzed Raman spectra of the central and ring regions of DCDs of ST, model tears of infectious ulcerative keratitis (IUK) composed of a mixture of ST and *Staphylococcus aureus*, as well as a mixture of ST and *Pseudomonas* aureus on Ti/Au coated glass slides based on PCA (Kuo et al., 2011). They found the spectra of the central region were more specific and could be used to effectively distinguish patients with IUK from patients with noninfectious central or paracentral ulcerative keratitis (NIUK) as shown in Figure 4A, demonstrating a promising prospect of tear DCDRS in the diagnosis of ocular surface infectious diseases. Xie et al. classified tear Raman spectra of patients with keratitis and healthy individuals using PCA-SVM and PLS-SVM models with an accuracy exceeds 77% (Xie et al., 2020). Wu et al. classified keratitis and conjunctivitis from healthy subjects using RS and deep learning models such as CNN and RNN with accuracies greater than 90%, indicating the great potential of RS of tears combined with depth learning algorithms in clinical screening of keratitis and conjunctivitis (Wu et al., 2022). Li et al. distinguished open-angle glaucoma (POAG), primary angle-closure glaucoma (PACG) patients, and a normal group using DCDRS of tears and pointed out the Raman spectroscopic features to reflect chemical information related to amino acids, proteins, and lipids in tears (Li et al., 2023b). The accuracy of classification of the samples using an SVM model based on PCA-LDA dimensionality reduction is over 90%, indicating the feasibility of using tear samples for glaucoma diagnosis. In addition to ophthalmic diseases, RS of tears can also be used for the diagnosis of neurodegenerative diseases since the biomarkers of neurodegenerative diseases in the systemic blood circulation can rejoin the vascularization of other body districts, as the retina, lens, and lacrimal regions, affecting the composition of tears. The spectral differences of tear SERS in Alzheimer’s disease patients (AD), mild cognitive impairment (MCI), and healthy control group (Ctr) were investigated as shown in Figure 4B (Cennamo et al., 2020). The average SERS spectra differences among Ctr, MCI, and AD subjects are related to lactoferrin and lysozyme protein components. To classify these three types, multiple spectral intervals from SERS spectra of tears were analyzed using PCA for feature extraction, and classification models were built using the naïve Bayes classification algorithm. The results showed all 31 samples were classified correctly, indicating great prospect of the proposed method as support of clinical diagnosis and discrimination of AD from other forms of dementia. Ami et al. discriminated between tears from amyotrophic lateral sclerosis patients and healthy controls with a high specificity of 100% and sensitivity of 100% using RS combined with mean partial least-square discriminant analysis (PLS-DA) as shown in Figure 4C (Ami et al., 2021). The potential of RS of tears combined with machine learning in the diagnosis of other diseases such as asymptomatic breast cancer, cerebral infarction, and jaundice has also been explored (Kim et al., 2020; Zhang et al., 2020; Fan et al., 2021; Zhao et al., 2022). The applications of RS of tears in disease diagnosis are summarized in Table 1. These researches demonstrate a variety of possibilities of RS of tears in disease diagnosis and the disease biomarkers revealed by the research results deserve further clarification for pathological analysis.

**TABLE 1:** Applications of Raman spectroscopy of tears in the diagnosis of diseases.

Diseases	RS techniques	Analysis methods	Samples	References
Heat-denatured herpes simplex virus (One of the causes of conjunctivitis)	SERS	LDA	ST, ST, and TM, ST and TM, HSV	Reyes-Goddard et al. (2008)
Adenoviral conjunctivitis	DCD-SERS	PCA	tears from patients and healthy subjects	Choi et al. (2014)
Ulcerative keratitis	DCD-SERS	PCA	STs, STs mixed with *Staphylococcus aureus*, and STs mixed with *Pseudomonas* aureus	Kuo et al. (2011)
Infectious keratitis	CRS	PCA-SVM, PLS-SVM	tears from patients and healthy subjects	Xie et al. (2020)
Keratitis and conjunctivitis	CRS	mRMR/PCA/PLS based CNN/RNN	tears from keratitis patients, conjunctivitis patients, and healthy subjects	Wu et al. (2022)
Glaucoma	DCDRS	PCA-LDA based SVM	POAG patients, PACG patients, and healthy subjects	Li et al. (2023b)
Alzheimer’s disease	DCD-SERS	PCA based Bayes	tears from patients and healthy subjects	Cennamo et al. (2020)
Amyotrophic lateral sclerosis	CRS	PLS-DA, extreme gradient boosting (xgbTree)	tears from patients and healthy subjects	Ami et al. (2021)
Asymptomatic breast cancer	SERS	PCA-LDA	tears from patients and healthy subjects	Kim et al. (2020)
Cerebral infarction	CRS	PCA-SVM	tears from patients and healthy subjects	Zhang et al. (2020)
Cerebral ischemia and cerebral infarction	CRS	PLS-PNN	ears from cerebral ischemia patients, cerebral infarction patients, and healthy subjects	Fan et al. (2021)
Jaundice	DCD-SERS	PCA-LDA	Tears from Jaundice-positive jaundice-negative patients	Zhao et al. (2022)

## 3 Conclusion and prospects

So far, RS has been successfully applied to ocular biofluid detections for non-invasive BG monitoring, ophthalmic pharmacokinetic analysis, characterization of tear components, and diagnosis of diseases. For non-invasive BG monitoring, clinically relevant glucose concentration was measured *in vitro* using calibration models based on RS of artificial AH. For ophthalmic pharmacokinetics analysis, various ophthalmic drugs such as ganciclovir, ceftazidime, AmB, ciprofloxacin, ketorolac tromethamine, and norepinephrine hydrochloride in AH were detected *in vitro* or *in vivo*. For the characterization of tear composition, high-sensitive methods such as DCDRS, SERS, and a combination of these two have been used to detect proteins, lipids, UA, hypoxanthine, bicarbonate, glucose, and drug components. For disease diagnosis, Raman spectra and machine learning methods have been used to diagnose ophthalmic diseases such as conjunctivitis, keratitis, glaucoma, neurodegenerative diseases, and other diseases. The rich applications of RS in ocular biofluids show the great potential of RS in ophthalmology.

However, most studies are still in the laboratory stage, and clinical applications of RS still face some challenges. Firstly, due to the low content of biomarkers in ocular biofluids, quantifying these micro or even trace components pose a challenge for Raman spectrum sensitivity. To detect AH components, it is necessary to ensure that the photothermal effect at the focal point of the incident light does not cause irreversible damage to the near tissues and the scattered light reaches the retina below the safety threshold. The low power level of the incident light also limits Raman scattering intensity. To detect tear components, the sensitivity of SERS alone for certain low-concentration proteins is still insufficient. DCDRS and the combination of SERS and DCDRS methods make it difficult to quantify specific analytes based on the spectral intensity of a certain point in the deposition area due to the non-uniform distribution of tear components. Secondly, since RS provides optical fingerprints of various chemical bond vibration information of Raman-active molecules, different molecules may contain the same functional groups, making it difficult to determine disease biomarker molecules directly through Raman signatures. For non-invasive detection of AH, Raman spectra may contain contributions from the tear film and cornea, and how to eliminate these interferences also poses challenges. Finally, the disease recognition ability and model generalization ability of RS combined with machine learning models must be further strengthened for clinical applications. Most existing models based on machine learning using RS for identifying ophthalmic diseases use smaller datasets, and the accuracy of models under larger training and testing sets needs to be verified. In response to the above three issues, the following prospects are made. Firstly, for the detection of different components, using incident light wavelengths with a higher Raman signal-to-noise ratio or RRS may be a potential method to improve the sensitivity of RS. For AH detection, the design and production of safe and low-loss eye probes to collect Raman light also show great potential in improving detection sensitivity (Zhang et al., 2021). For the detection of tears, SERS based on aggregators such as aluminum or magnesium ions, its combination with microfluidic technology, and special probes that can collect more Raman scattering light such as photonic crystal fiber probes may be the answer to improving detection sensitivity and repeatability (Inscore et al., 2011; Yang, et al., 2012; Wu et al., 2014; Bao et al., 2020). Secondly, utilizing neural networks to distinguish the contributions from different components and spectral features from different biofluids and organizations is expected to make more accurate biomolecular recognition and quantification possible. For AH detection, a self-organising map discriminant index (SOMDI) method may be a potentially effective approach to distinguish the spectral features of tear film and cornea to obtain spectral information only from AH (Banbury et al., 2019). Finally, with the development and maturity of optoelectronic devices, miniaturized and low-cost RS devices are expected to be more applied in clinical research, and obtaining more sample data becomes a driving force for improving the performance of artificial intelligence models. In summary, RS technology still has great potential for development in the detection of ocular biofluids and further eye tissue components, and this technology is expected to bring new changes to the diagnosis of ophthalmic diseases.
